# Gastrointestinal Stability and Cytotoxicity of Bacteriocins From Gram-Positive and Gram-Negative Bacteria: A Comparative *in vitro* Study

**DOI:** 10.3389/fmicb.2021.780355

**Published:** 2022-01-25

**Authors:** Samira Soltani, Séverine Zirah, Sylvie Rebuffat, Frédéric Couture, Yvan Boutin, Eric Biron, Muriel Subirade, Ismail Fliss

**Affiliations:** ^1^Food Science Department, Food and Agriculture Faculty, Laval University, Quebec, QC, Canada; ^2^Muséum National d’Histoire Naturelle, Centre National de la Recherche Scientifique, Laboratory Molecules of Communication and Adaptation of Microorganisms, UMR 7245 CNRS-MNHN, Paris, France; ^3^TransBIOTech, Lévis, QC, Canada; ^4^Faculty of Pharmacy, Laval University, Quebec, QC, Canada; ^5^Institute of Nutrition and Functional Foods, Laval University, Quebec, QC, Canada

**Keywords:** bacteriocins, *in vitro* digestion, cytotoxicity, hemolysis, food preservative

## Abstract

Bacteriocins are receiving increased attention as potent candidates in food preservation and medicine. Although the inhibitory activity of bacteriocins has been studied widely, little is known about their gastrointestinal stability and toxicity toward normal human cell lines. The aim of this study was to evaluate the gastrointestinal stability and activity of microcin J25, pediocin PA-1, bactofencin A and nisin using *in vitro* models. In addition cytotoxicity and hemolytic activity of these bacteriocins were investigated on human epithelial colorectal adenocarcinoma cells (Caco-2) and rat erythrocytes, respectively. Pediocin PA-1, bactofencin A, and nisin were observed to lose their stability while passing through the gastrointestinal tract, while microcin J25 is only partially degraded. Besides, selected bacteriocins were not toxic to Caco-2 cells, and integrity of cell membrane was observed to remain unaffected in presence of these bacteriocins at concentrations up to 400 μg/mL. In hemolysis study, pediocin PA-1, bactofencin A, and nisin were observed to lyse rat erythrocytes at concentrations higher than 50 μg/mL, while microcin J25 showed no effect on these cells. According to data indicating gastrointestinal degradation and the absence of toxicity of pediocin PA-1, bactofencin A, and microcin J25 they could potentially be used in food or clinical applications.

## Introduction

Bacteriocins are gene-encoded antimicrobial peptides produced by Gram-positive and Gram-negative bacteria with a broad or narrow spectrum of inhibition, often targeting closely related strains. Bacteriocins have a broad diversity in terms of sizes, structures, and mechanisms of action (Cotter et al., 2013; Yang et al., 2014). Our team developed a database called BACTIBASE^1^ which provides vital information on biochemical properties and spectrum of inhibition activity of different bacteriocins produced by both Gram-positive and Gram-negative bacteria including those considered in this study (Hammami et al., 2010). Rapid identification of bacteriocins and their unique characteristics have drawn increasing attention toward their application as food preservatives (O’Connor et al., 2020) or therapeutic agents (Cotter et al., 2013). Bacteriocins can be excellent candidates to enhance food safety and quality by replacing the frequent use of chemical preservatives, many of which have been shown to exhibit toxicity (Kumar et al., 2019). Bacteriocins are tasteless, odorless, and colorless peptides which can be incorporated into foods without interfering with their organoleptic properties (Perez et al., 2014). In addition, bacteriocins have been shown to be stable against heat, extreme pH, and high salt concentrations. Inhibitory effects of bacteriocins from Gram-positive bacteria, especially lactic acid bacteria (LAB), against food pathogens and spoilage microorganisms is well documented. Several studies have reported the application of bacteriocins as preservatives in different food matrices such as meat, dairy products, fermented vegetables, beverages, etc. (Gálvez et al., 2011; Hassan et al., 2021). But, despite the bio-preservative potential of bacteriocins, nisin is the only bacteriocin approved to be used as a food additive by regulatory agencies, including the World Health Organization (WHO)/Food Development Authority (FDA, United States), and the European Food Safety Authority (ESFA) (European Commission [EC], 1983; FDA, 1988). There are different commercial products of nisin, such as Nisaplin^®^ (Danisco, Copenhagen, Denmark), Nisin Z^®^ (Handary, Brussel, Belgium), and Delvo^®^ Nis (DSM, Delft, Netherlands).

Although bacteriocins were mainly studied as food additives, they have exhibited desirable properties for clinical applications as well. Interestingly, several bacteriocins were found to be effective against multi-drug-resistant bacterial strains; hence, they can be used in human and animals for treating local or systemic infections caused by antibiotic-resistant bacteria (Cotter et al., 2013). In fact, the narrow inhibition spectrum of bacteriocins, their specific modes of action, high potency, ability to be bioengineered and reduced risk of resistant development, qualify them to be considered as promising alternatives to conventional antibiotics. Moreover, their narrow spectrum of activity enables them to shape gut microbiota in human and animals (Heilbronner et al., 2021).

However, although bacteriocins have shown great potential for clinical applications, only a few bacteriocins have been progressed to be used in clinical trials. Those include microbisporicin (NAI-107, Naicons SRL and Sentinella Pharmaceuticals), mutacin 1140 (MU1140 Oragenics, United States) and duramycin (Moli1901, AOP Orphan Pharmaceuticals and Lantibio) (Sandiford, 2015).

In the veterinary sector, due to the increased emergence of antibiotic-resistant strains, the systemic use of antibiotics as growth promoters has been banned in many countries. Consequently, bacteriocins or their producing strains can be considered as promising safe alternatives in order to target pathogens and improve animal health. Microcin J25 is a Gram-negative bacteriocin and it has been used to control *Salmonella* in poultry and improve growth performance in chicken (Wang et al., 2020) and pig (Yu et al., 2017). Additionally, nisin-based products such as Mast Out^®^, Teatseal^®^, Wipe Out^®^ (FDA approved wipe) are commercially available for mastitis treatment.

Although most of the bacteriocin-producing strains possess the Generally Recognized as Safe (GRAS) status (FDA, 1988; Alvarez-Sieiro et al., 2016), the use of bacteriocins in human and animal applications requires rigorous evaluation of their safety and efficacy by different *in vitro* studies on eukaryotic cells followed by *in vivo* studies on animal models (Soltani et al., 2021b). Although the effectiveness of bacteriocins against important pathogens and spoilage organisms has been well documented (Galvez et al., 2008; Cotter et al., 2013), there is very limited data available on their gastrointestinal (GI) stability or acute and chronic toxicity. Over years, the GI behavior of bacteriocins has become an important research topic. Indeed, pediocin PA-1 was shown to be sensitive to GI conditions (Kheadr et al., 2010), while microcin J25 remained quite stable during the GI transit (Naimi et al., 2018). It should be noted that the safety criteria concerning the use of bacteriocins may vary based on their intended application. For instance, as food additives, bacteriocins are crucial to be hydrolyzed while passing through the GI tract, whereas this attribute might not be favorable for the therapeutic use. Therefore, bacteriocins might get approved by regulatory agencies according to their intended use, either as food additives or technological agents. As technological agents, no toxicity assessment is required upon degradation during the GI passage (Soltani et al., 2021b). On the other hand, as food additives, bacteriocins must be subjected to a complete battery of tests.

In this study we examined the GI physicochemical stability and toxicity of four highly purified bacteriocins representing different classes of bacteriocins and having different structures and mechanisms of action. Namely, two ribosomally synthesized and posttranslationally modified peptides (RiPPs), nisin (lantibiotic; class I bacteriocin) and microcin J25 (lasso peptide), and two non-modified bacteriocins pediocin PA-1 (class IIa bacteriocin) and bactofencin A (class IId bacteriocin) were selected. In addition to their comportment in conditions miming those of the GI tract, their cytotoxicity on human colonic adenocarcinoma cells and hemolytic activity were evaluated.

## Materials and Methods

### Bacterial Strains and Culture Condition

Microcin J25 was produced by *Escherichia coli* MC4100 carrying the plasmid PTUC 202 (Solbiati et al., 1999). For the antibacterial activity of pure compounds and digestion mixture, *Listeria ivanovii* HPB28 (Canada Health Protection Branch) was used as indicator strain for nisin and pediocin PA-1, while *Staphylococcus aureus* ATCC 6538 (ATCC) and *Salmonella enterica* subsp. *enterica* serovar Newport ATCC 6962 (later referred to as S. Newport, ATCC) were used as indicator strains for bactofencin A (M14L, M18L) and microcin J25, respectively. *L. ivanovii* HPB28 and *S. aureus* were cultured in Tryptic Soy (Difco Laboratories, Spark, MD, United States) supplemented with 0.6% yeast extract (TSBYE), at 30 and 37°C, respectively. S. Newport was cultured at 37°C overnight in Luria-Bertani (LB) (Difco Laboratories, Spark, MD, United States). All strains were maintained at –80°C as stock cultures in their corresponding culture media supplemented with 20% glycerol, and propagated twice at 24 h intervals before use.

### Production and Purification of Bacteriocins

#### Microcin J25

Production and purification of microcin J25 were carried out as described previously (Naimi et al., 2018). Briefly, minimal medium (M63) containing KH_2_PO_4_ (3 g/L), K_2_HPO_4_ (7 g/L), (NH_4_)_2_HPO_4_ (2 g/L), and casamino acid (1 g/L) was supplemented with 1 mL/L of 20% MgSO_4_, 10 mL/L of 20% glucose, and 1 mL/L of 1 g/L thiamine, inoculated with an overnight culture of *E. coli* MC4100 pTUC202 (2% v/v in LB broth), followed by overnight incubation at 37°C in rotary shaking at 250 rpm. Bacterial cells were separated by centrifugation at 8,000 *g* for 20 min at 4°C. The supernatant was pre-purified by solid phase extraction (Sep-Pak C18) at 4°C at a flow rate of 2 mL/min, followed by reversed-phase high-performance liquid chromatography (RP-HPLC, Beckman Coulter System Gold Preparative HPLC system, Mississauga, ON, Canada) on a preparative C18 column (Luna 10 μm, 250 mm x 21.10 mm, Phenomenex, CA, United States) at a flow rate of 10 mL/min. Microcin J25 was quantified by RP-HPLC using an analytical C18 column (Aeris 3.6 μm, PEPTIDE XB-C18, 250 Î 4.6 mm, Phenomenex, CA United States) (Naimi et al., 2018).

#### Pediocin PA-1(M31L)

Pediocin PA-1 carrying the Met31Leu substitution [pediocin PA-1(M31L)], was selected for the study, as the antimicrobial activity of this linear analog is similar to the wild-type bacteriocin naturally produced by *Pediococcus acidilactici*, while its stability is improved by the lack of Met31which is sensitive to oxidation. Briefly, pediocin PA-1(M31L) was synthesized as described previously (Bédard et al., 2018) by standard solid phase peptide synthesis (SPPS) on prelude peptide synthesizer from Gyros Protein Technologies (Tucson, AZ, United States) using HMBP-ChemMatrix^®^ resin. The peptide purification was carried out by RP-HPLC with a Shimadzu Prominence system on a Phenomenex Kinetex^®^ EVO C18 column (250 mm × 21.2 mm, 300 Å, 5 μm) and UV detection at 220 and 254 nm using 0.1% AcOH/H_2_O (A) and 0.1% AcOH/CH_3_CN (B), at 14 mL/min flow rate.

#### Bactofencin A(M14L, M18L)

Similar to pediocin PA-1, a linear analog of bactofencin A was produced as described previously by Beìdard et al. (2018). The methionine residues were replaced with leucines in bactofencin A(M14L, M18L) analog. Similarly, this analog of bactofencin A showed the same potency as the bacteriocin produced naturally by *Lactobacillus salivarius*, while its stability was enhanced (Beìdard et al., 2018).

#### Nisin Z

The commercial preparation containing 10% nisin Z was purchased from Niseen, chemical, United States. Purification was performed by the salting out method as described by Gough et al. (2017). Nisin was quantified by RP-HPLC on the analytical C18 column (Aeris 3.6 μm, PEPTIDE XB-C18, 250 mm × 4.6 mm, Phenomenex, CA, United States).

### *In vitro* Simulated GI Digestion

*In vitro* simulated oral, gastric, and small intestinal digestion was adopted from standardized INFOGEST protocol (Brodkorb et al., 2019) in three independent replicates. The initial concentration of bacteriocins was selected in order to have sufficient quantity in the digestion mixture to conduct both activity assay by agar well diffusion and by RP-HPLC for quantification.

The entire digestion procedure was performed at 37°C.

*Oral conditions*: 5 mL of a 8 mg/mL bacteriocin solution (in water) were mixed with simulated salivary fluid [SSF: KCl 15.1 mM, KH_2_PO_4_ 3.7 mM, NAHCO_3_ 13.6 mM, MgCl_2_(H_2_O)_6_ 0.15 mM, (NH_4_)_2_CO_3_ 0.06 mM, HCl 1.1mM, CaCl_2_ (H_2_O)_2_ 1.5 mM] in 1:1 (v/v) ratio, and the final solution was incubated for 2 min at 37°C.

*Gastric conditions*: The sample from the oral condition was diluted in 1:1 (v/v) ratio with simulated gastric fluid [SGF: KCl 6.9 mM, KH_2_PO_4_ 0.9 mM, NAHCO_3_ 25 mM, NaCl 47.2 mM, MgCl_2_(H_2_O)_6_ 0.12 mM, (NH_4_)_2_CO_3_ 0.5 mM, HCl 15.6 mM, CaCl_2_ (H_2_O)_2_, 0.15 mM] containing pepsin (2000 U/mL in the gastric mixture), and lipase (60 U/mL in the gastric mixture). The final solution (final volume of 20 mL) was then adjusted to pH 3 with HCl 5 M, followed by incubation under shaking for 90 min at 37°C.

*Small intestine conditions*: Samples from gastric conditions were diluted in 1:1 (v/v) ratio with 20 mL of simulated intestinal fluid[SIF KCl 6.8 mM, KH_2_PO_4_ 0.8 mM, NAHCO_3_ 85 mM, NaCl 38.4 mM, MgCl_2_(H_2_O)_6_ 0.33 Mm, HCl 8.4 mM, CaCl_2_ (H_2_O)_2_, 0.6 mM] containing bile salt (10 mmol/L in the final gastric mixture) and pancreatin (100 U/mL in final digestion mixture) to acquire a final volume of 40 mL. The final solution was adjusted to pH 7 using NaOH 5 M, followed by incubation for a further 2 h at 37°C. The digestion mixtures were heat-treated at 80°C for 10 min and centrifuged at 8,000 *g* for 10 min at 4°C for further analysis. Bacteriocins in the different samples were analyzed by analytical HPLC-UV and liquid-chromatography mass spectrometry (LC-MS/MS). The antimicrobial activity of the samples was also estimated by microtitration assays and agar well diffusion assays.

### Antimicrobial Activity Assays

#### Agar Well Diffusion Assays

The inhibitory activity of the different bacteriocins was determined qualitatively by the agar well diffusion assay, as described by Bédard et al. (2018). Briefly, 80 μL of bacteriocin samples were added into wells punched out in appropriate media (25 mL) seeded with 250 μL of overnight culture of indicator strains. Following an 18 h incubation at the appropriate temperature, inhibition zones were measured. Pictures were taken by ChemiDoc XRS (Bio-Rad, Hercules, CA, United States).

#### Microdilution Assay

Quantitative determination of inhibitory activity of bacteriocins was carried out using the broth microdilution method as described by Ben Said et al. (2020). Two-fold serial dilutions of bacteriocins (125 μL) were prepared in an appropriate medium in a clear 96-well flat-bottom microtiter plate. Indicator strains were diluted to 10^5^ CFU/mL, from which 50 μL were added to each well. The microtiter plates were incubated for 24 h under appropriate conditions, and optical densities were recorded at 595 nm (Infinite M200, Tecan, Switzerland). Control wells contained untreated culture and appropriate medium (blanks). The number of inhibition wells was noted and inhibition activities were calculated in μg/mL.

### Analysis of Samples by UHPLC-MS/MS and Molecular Networking

The digestion mixtures were analyzed by LC-MS/MS on an ultra-high-performance LC system (Ultimate 3000 RSLC, Thermo Fisher Scientific) connected to high-resolution electrospray ionization – quadrupole – time of flight (ESI-Q-TOF) mass spectrometer (Maxis II ETD, Bruker Daltonics). Separations were achieved on an Acclaim RSLC Polar Advantage II column (2.2 μm, 2.1 mm × 100 mm, Thermo Fisher Scientific) at a flow rate of 300 μL/min, using the following gradient of solvent A (ultra-pure water/0.1% formic acid) and solvent B (HPLC-MS grade acetonitrile/0.08% formic acid) over a total run time of 17.5 min: linear increase from 10% B to 60% B for 12 min, linear increase to 100% B for 0.2 min, decrease to 10% B for 0.5 min. The ESI-Q-TOF instrument was externally calibrated before each run using a sodium formate solution consisting of 10 mM sodium hydroxide in isopropanol/0.2% formic acid (1:1, v/v). Data-dependent LC-MS/MS data were acquired in positive ion mode in the mass range m/z 250–2500, using collision induced dissociation with collision energy calculated from m/z and charge states. The LC-MS/MS data were treated with Data Analysis 4.4 (Bruker Daltonics).

The liquid-chromatography mass spectrometry (LC-MS/MS) data were converted into mgf files and subjected to the online GNPS workflow^2^ (Yang et al., 2013; Wang et al., 2016), using the following set-up: parent ion mass tolerance of 0.05 Da, fragment ion mass tolerance 0.05 Da, cluster minimal size 1, minimum matched peaks 4 and minimum cosine similarity score 0.5. The resulting networks were visualized using Cytoscape 3.8.2. The identification of bacteriocin degradation products from MS/MS data was performed by focussing on the clusters containing only nodes absent in the control with no bacteriocin. Peptide assignment was performed by database search using PEAKS Studio 10.6 using the following parameters: parent and fragment mass error tolerance: 0.05 Da, enzyme: none, digest mode: unspecific, variable modifications: oxidation (M), deamidation (NQ), dehydration (S/T, for nisin Z only), dehydrogenation (C, to account for thioether or disulfide bond), amidation [Cter, for bactofencin, bactofencin A(M14L, M18L)], max variable post-translational modification (PTM) per peptide: 5–6. The search was performed against a homemade database composed of the bacteriocin sequences. A false discovery rate (FDR) of 1% was set to peptide spectrum match and protein levels. For nisin Z, which contains more complex PTMs, assignments were also proposed by Mw calculations using ChemBioDraw Ultra version 12.0.2.1076. Molecular networking and PEAKS identifications were crossed and validated by manual inspection of the MS/MS data.

### Cell Culture

The human colonic adenocarcinoma (Caco-2) cells were purchased from ATCC. Cells were cultured in DMEM medium in a humidified atmosphere containing 5% CO_2_ at 37°C. For the LDH release assay, cells were seeded in 96 well flat bottom culture plates (1 × 10^4^ cells/well). Caco-2 cells, which have been frequently used for permeability studies across the intestinal epithelium, were selected in this study for evaluating the cytotoxicity of bacteriocins and their interaction with intestinal epithelium during GI transit to determine their cytotoxicity.

### *In vitro* Cytotoxicity Assay

#### LDH Release Assay

The CytoTox-ONE™ cytotoxicity assay kit (Promega, United States) was used to measure the LDH release. The growing Caco-2 cells (in DMEM + 10% FBS) were treated with 100 μL of bacteriocins at different concentrations and incubated at 37°C for 48 h with 5% CO_2_. Lysis solution (Promega) was added in a 1:1 ratio to selected control wells to induce the maximal release of LDH and the plate was incubated for 5 min. Then 100 μL of medium were transferred to another 96-well plate for LDH release measurement and CytoTox-ONE™ reagent was added to each well and incubated for 10 min at room temperature. Finally, stop solution was then added to each well, and absorbance was measured using a spectrophotometer (Tecan Spark 20M, Morrisville, NC, United States) at excitation/emission wavelengths of 560/590 nm. Each compound-treated value was blanked with the values of the control-treated cells and cytotoxicity was expressed as percentage of the maximal LDH release (lysis solution treated cells), calculated by the following formula:


$$\%\,C\,y\,t\,o\,t\,o\,x\,i\,c\,i\,t\,y=\frac{\begin{array}{c} L\,D\,H\,a\,c\,t\,i\,v\,i\,t\,y\,o\,f\,c\,e\,l\,l\,s\,t\,r\,e\,a\,t\,e\,d\,w\,i\,t\,h\,c\,o\,m\,p\,o\,u\,n\,d-\\ S\,p\,o\,n\,t\,a\,n\,e\,o\,u\,s\,L\,D\,H\,a\,c\,t\,i\,v\,i\,t\,y \end{array}}{\begin{array}{c} M\,a\,x\,i\,m\,u\,m\,L\,D\,H\,a\,c\,t\,i\,v\,i\,t\,y-S\,p\,o\,n\,t\,a\,n\,e\,o\,u\,s\\ L\,D\,H\,a\,c\,t\,i\,v\,i\,t\,y \end{array}}$$

#### Hemolytic Activity

The experiment was performed after prior approval from the local ethics committee by TransBIOTech laboratory. Hemolytic potential of bacteriocins was evaluated according to FDA (2005). Rat blood was collected into a heparinized tube and added in a 1:1 volume (100 μL) to serially diluted compounds (in 100 μL PBS), in a conical 96-well plate. 10% Triton X-100 and PBS were used as a positive and negative controls, respectively. The plates were sealed and incubated for 45 min at 37°C prior to centrifugation at 2,000 *g* for 10 min to pellet red blood cells. The supernatant was then transferred into clear 96-well plates and the absorbance was read at 540 nm (hemoglobin). The percentage of hemolysis was calculated as follow, with RBC standing for red blood cells:


$$\%\,H\,e\,m\,o\,l\,y\,s\,i\,s=\frac{\begin{array}{c} A\,b\,s\,o\,r\,b\,a\,n\,c\,e\,o\,f\,R\,B\,C\,t\,r\,e\,a\,t\,e\,d\,w\,i\,t\,h\,b\,a\,c\,t\,e\,r\,i\,o\,c\,i\,n\,s\\ -A\,b\,s\,o\,r\,b\,a\,n\,c\,e\,o\,f\,R\,B\,C\,t\,r\,e\,a\,t\,e\,d\,w\,i\,t\,h\,P\,B\,S \end{array}}{\begin{array}{c} A\,b\,s\,o\,r\,b\,a\,n\,c\,e\,o\,f\,R\,B\,C\,t\,r\,e\,a\,t\,e\,d\,w\,i\,t\,h\,T\,r\,i\,t\,o\,n\,X\,100\\ -A\,b\,s\,o\,r\,b\,a\,n\,c\,e\,o\,f\,R\,B\,C\,t\,r\,e\,a\,t\,e\,d\,w\,i\,t\,h\,P\,B\,S \end{array}}$$

### Statistics

Data were expressed as mean ± standard deviation (SD) for at least three independent experiments. Dose-response curves were generated using GraphPad Prism version 8.2 GraphPad Software (San Diego, CA, United States).

## Results

### Bacteriocin Production

**FIGURE 1:**
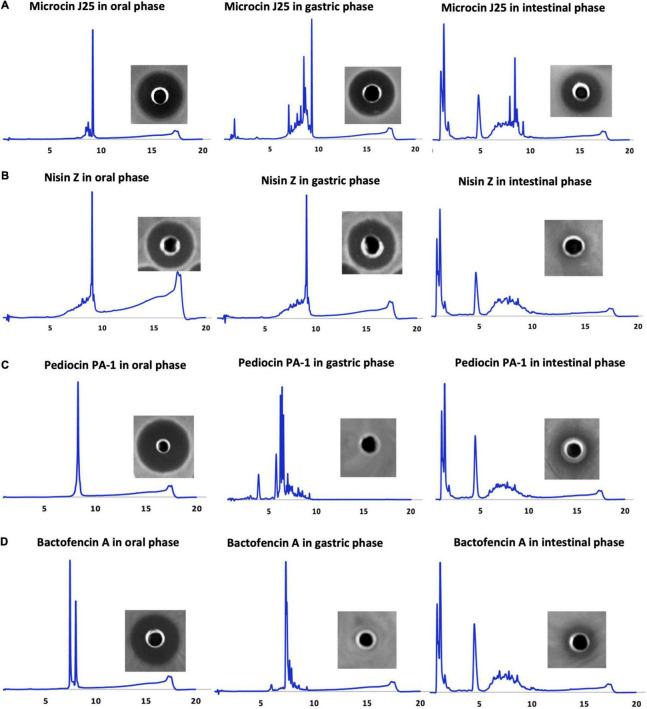
HPLC profiles of oral, gastric, and small intestinal digestion conditions of **(A)** microcin J25, **(B)** nisin Z, **(C)** pediocin PA-1(M31L), and **(D)** bactofencin A (M14L, M18L), along with their respective inhibitory activities against indicator strains obtained after incubation period (microcin J25 against *S.* Newport ATCC 6962, nisin Z and pediocin PA-1 against *L. ivanovii* HPB28, bactofencin A against *S. aureus* ATCC 6538).

Highly pure pediocin PA-1(M31L), nisin Z, bactofencin A(M14L, M18L) and microcin J25 were produced (Figure 1). The MS spectra of the purified peptides after GI digestion are provided as Supplementary Figures 1–4. For bactofencin A(M14L, M18L), two peaks were detected, one corresponding to the native peptide and the second one to the reduced form (+2 Da). Pediocin PA-1(M31L) was detected in its reduced form (+4 Da).

Such a high purity level is a prerequisite for *in vitro* GI stability and toxicity studies. The antimicrobial activity of the bacteriocins was confirmed by agar diffusion assays, which showed significant inhibition against target bacteria.

### Stability and Antibacterial Activity of Bacteriocins in GI Conditions

The physiochemical and biological stability of the bacteriocins in GI tract conditions was assessed using RP-HPLC, microdilution, and agar well diffusion assay (Figure 1 and Table 1). Gastrointestinal stability of microcin J25 was similar to our previous study by Naimi et al. (2018), hence MS and molecular network of microcin J25 are not shown here. All the bacteriocins were observed to be stable and retain their inhibitory activity in oral conditions. In gastric conditions, despite of high acidic pH, nisin Z retained its activity with minor degradation peaks, indicating good gastric stability; however, under small intestinal conditions, it appeared to be significantly degraded, indicating the loss of its inhibitory activity (Figure 1A). Pediocin PA-1(M31L) and bactofencin A(M14L, M18L) were observed to be significantly degraded in gastric condition, leading to their significant loss of activity (Figures 1C,D). It should be noted that disulfide bridge in pediocin PA-1(M31L) and bactofencin A(M14L, M18L) are formed spontaneously in buffered aqueous media where cyclization was observed. This may explain the presence of two peaks of bactofencin A(M14L, M18L) in HPLC chromatogram.

**TABLE 1:** Total activity of microcin J25, nisin Z, pediocin PA-1(M31L), and bactofencin A(M14L, M18L) against indicator strains (S. Newport ATCC 6962, *L. ivanovii* HPB 28, *S. aureus* ATCC 6538) according to the different conditions used at the oral, gastric, small intestinal levels, and pure compounds without GI digestion.

Samples	AU/mL	Strain
Microcin J25	0.0712	*S.* Newport ATCC 6962
Microcin J25 oral	0.0712	
Microcin J25 gastric	0.0712	
Microcin J25 intestinal	0.356	
Nisin Z	3.12	*L. ivanovii* HPB 28
Nisin Z oral	3.12	
Nisin Z gastric	3.12	
Nisin Z intestinal	200	
Pediocin PA-1	0.18	*L. ivanovii* HPB 28
Pediocin PA-1 oral	0.18	
Pediocin PA-1 gastric	–	
Pediocin PA-1 intestinal	–	
Bactofencin A	10.4	*S. aureus* ATCC 6538
Bactofencin A oral	10.4	
Bactofencin A gastric	–	
Bactofencin A intestinal	–	

### LC-MS Analysis

**FIGURE 2:**
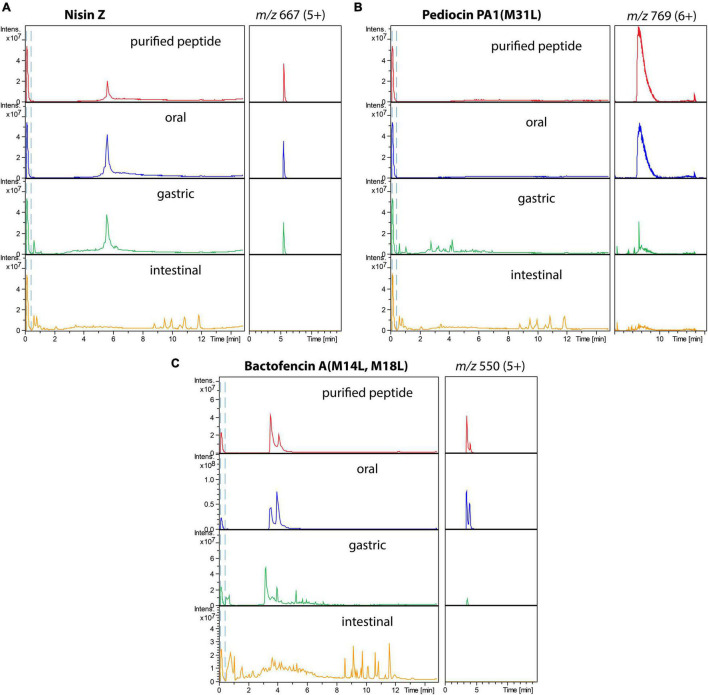
Total ion chromatograms (left) and extracted ion chromatograms of the major ion (right) of purified bacteriocins (in red), bacteriocins incubated in oral (blue), gastric (green), and small intestinal (orange) conditions: **(A)** nisin Z, **(B)** pediocin PA-1(M31L), **(C)** Bactofencin A (M14L, M18L).

The stability of the three bacteriocins in the different GI conditions was further studied using LC-MS (Figure 2). Analysis of LC-MS confirmed that all bacteriocins were very stable in oral digestion conditions, while degradation increased in the gastric [for pediocin PA-1(M31L) and bactofencin A(M14L, M18L)] and intestinal (for all three peptides) conditions.

**FIGURE 3:**
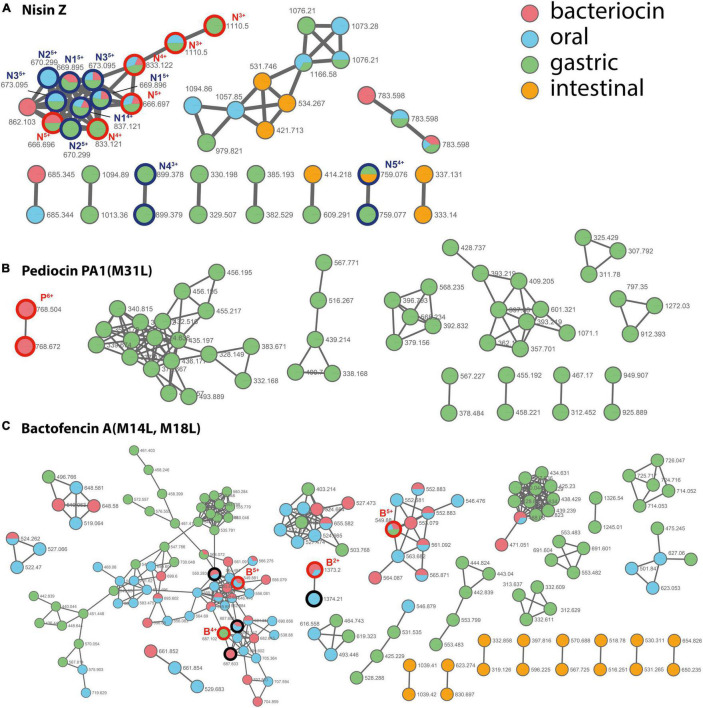
Molecular network of the nodes detected for bacteriocins (in red) and their degradation products after incubation in oral (in blue), gastric (in green), and small intestinal (in orange) digestion conditions. **(A)** nisin Z, **(B)** pediocin PA-1(M31L), **(C)** bactofencin A(M14L, M18L). The whole networks obtained for each bacteriocin are provided as Supplementary Figures 5–7. The nodes assigned to the intact bacteriocins are circled in bold red and annotated N, P, and B for nisin Z, pediocin PA-1(M31L), and bactofencin A(M14L, M18L), respectively, with charge state indicated as uppercase. The nodes assigned for nisin are circled in bold dark blue. Their assignment is provided as Supplementary Table 1. For bactofencin, the nodes assigned to bacteriocin with reduced disulfide bridge are circled in bold black.

Molecular networking derived from LC-MS/MS analysis of the digestion solutions allowed to assess the bacteriocin degradome in each condition (Figure 3 and Supplementary Figures 5–7). As a result of hydrolysis in the oral, gastric, and small intestinal conditions, nisin Z and bactofencin A(M14L, M18L) showed the lowest and highest number of degradation products, respectively. Degradation products of pediocin PA-1(M31L) were identified only in gastric conditions, which might be due to the large mass of this bacteriocin, suppressing the ionization of the intact and poorly hydrolyzed peptide in the LC-MS/MS conditions.

For nisin Z, a few degradation products were identified (Figure 3A and Supplementary Table 1). They result from cleavages at Ala28-Ser29, His31-Val32, and Lys12-Abu13. In addition, oxidized forms of the peptide were detected, together with ions with a +18 Da increment, which are proposed to result from hydrolysis in a thioether ring. For pediocin PA-1(M31L), the degradation products formed in gastric conditions resulted from multiple hydrolyses (Supplementary Figure 8). The first disulfide bridge (Cys9–Cys14) revealed a higher stability than the second one (Cys24–Cys44). Only short fragments located in the C-terminal region were detected after incubation in small intestine conditions. For bactofencin A(M14L, M18L), extensive fragmentation in the N-terminal region were revealed upon incubation in oral conditions, while the gastric medium yielded cleavages mainly in the C-terminal region, at Leu14 and Leu18 (Supplementary Figure 9).

### Cytotoxicity of Bacteriocins

**FIGURE 4:**
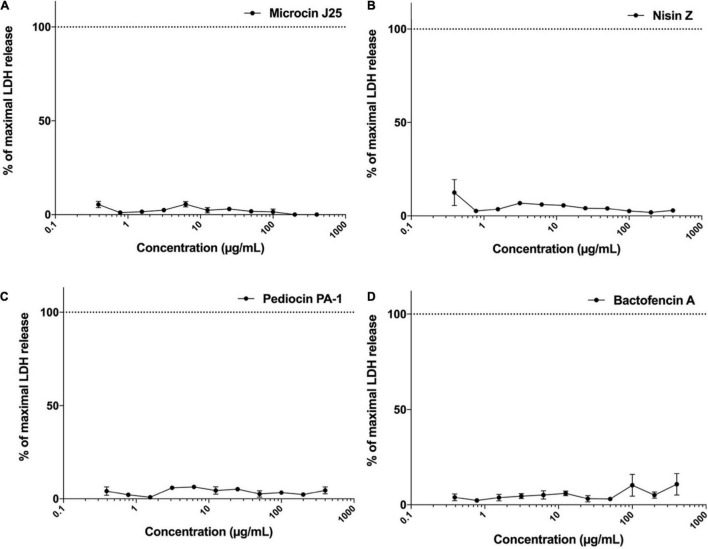
Effect of **(A)** microcin J25, **(B)** nisin Z, **(C)** pediocin PA-1(M31L), **(D)** bactofencin A (M14L, M18L) on LDH release (% control) at concentration range of 0.4–400 μg/mL. Data shown represent the mean values of three experiments ± SD.

The effect of different bacteriocins on the membrane integrity of Caco-2 cells was evaluated by LDH release assay. The dose-response curves for LDH release in Caco-2 cells treated with different concentrations of bacteriocins was plotted (Figures 4A–D). The results demonstrated that membrane integrity remained uncompromised in Caco-2 cells exposed to microcin J25, nisin Z, pediocin PA-1(M31L) and bactofencin A(M14L, M18L) at concentrations ranging from 0.4 to 400 μg/mL for 24 h. The maximal LDH release percentage was observed to be negligible in the medium as compared to control cells. It should be noted that the highest concentration tested (400 μg/mL) corresponds to approximately 10,000, 4,000, 40 and 400 times the MIC values of microcin J25, pediocin PA-1(M31L), bactofencin A(M14L, M18L) and nisin Z, respectively.

### Hemolytic Potential of Bacteriocins

The hemolytic activity of microcin J25, nisin Z, pediocin PA-1(M31L) and bactofencin A(M14L, M18L) was evaluated using rat erythrocytes as described previously (Soltani et al., 2021a). Results were expressed as percentage of hemolysis calculated by measuring the released hemoglobin after exposure to each bacteriocin at different concentrations, ranging from 0.4 to 400 μg/mL for 45 min.

**FIGURE 5:**
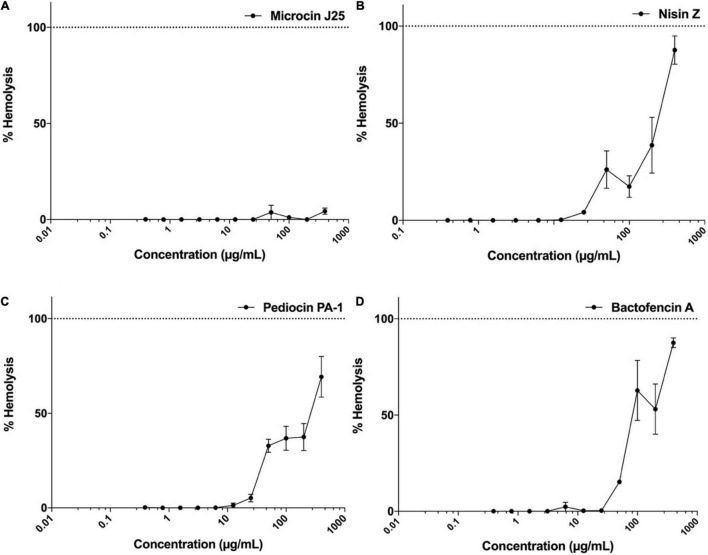
Hemolytic activity of **(A)** microcin J25, **(B)** nisin Z, **(C)** pediocin PA-1(M31L), **(D)** bactofencin A (M14L, M18L) at concentration range of 0.4–400 μg/mL. Data shown represent the mean values of three experiments ± SD.

The resulting dose-response curves are shown in Figures 5A–D. In the case of microcin J25, no hemolytic activity was observed at concentrations up to 400 μg/mL, while nisin Z, pediocin PA-1(M31L) and bactofencin A(M14L, M18L) showed a dose-dependent increase in the percentage of hemolysis at concentrations higher than 50 μg/mL. Both nisin Z and bactofencin A(M14L, M18L) showed approximately 100% hemolysis at concentrations close to 400 μg/mL.

## Discussion

Although bacteriocins have been recognized as potent antimicrobial agents with potential application in food, veterinary and clinical settings, they have remained underused. Since their discovery, bacteriocins have been widely studied as bio-preservatives; however, nisin has been the only legally approved bacteriocin used as a food additive. Additionally there are several reports on the *in vitro* efficacy of bacteriocins against clinically important pathogens, such as vancomycin-resistant *Enterococcus*, methicillin-resistant *Staphylococcus aureus* (MRSA), multidrug-resistant *Salmonella* and *E. coli* (Beìdard et al., 2018; Yu et al., 2019). Nonetheless, very few bacteriocins are entering the clinical pipeline. One of the key factor limiting the use of bacteriocins at an extended level could be insufficient data regarding their behavior in animal or human GI. Another important factor could be the lack of data regarding their possible toxicity and side effects as therapeutic agents (Soltani et al., 2021b).

In the current study, GI stability, cytotoxicity and hemolytic activity of different purified bacteriocins, either unmodified peptides or post-translationally modified peptides (nisin Z, microcin J25) have been determined using various well-accepted conventional *in vitro* models. When used as a food additives, the demonstration that the bacteriocin is degraded at the GI level is indicative of its non-toxicity since it neither gets absorbed nor comes into contact with the colonic microbiota; therefore, no adverse effect is created. However, if bacteriocin remains stable at GI level, it is imperative to provide the necessary data as an evidence of its non-toxicity to different cellular systems of the GI tract. Thus, the knowledge of bacteriocin stability in GI tract is crucial for its approval as a food preservatives. For medical and veterinary applications, the route of bacteriocin administration is determined based on its resistance to the various GI tract barriers, including low stomach pH and presence of numerous small intestinal proteolytic enzymes. The unstable bacteriocins are needed to be protected against GI tract conditions; thus, bioengineering and encapsulation technology have been developed to overcome such limitations. Considering the fact that the target site of most of the bacteriocins is in the colon, encapsulation technology has been implemented for controlled delivery and protection of these molecules against digestive enzymes (Gomaa et al., 2017; Gough et al., 2018).

There are limited number of studies regarding the stability of bacteriocins in the GI tract. It has been shown that bacteriocins can be degraded by proteolytic enzymes such as pepsin, trypsin and chymotrypsin in the stomach or intestine (Fernandez et al., 2013). Notably, class II bacteriocins are highly sensitive to intestinal proteases (Gough et al., 2017). In a human GI tract simulated *in vitro* model, pediocin PA-1 was observed to be stable in the stomach, but completely degraded in the small intestine (Kheadr et al., 2010). However, in the current study, pediocin PA-1(M31L) and bactofencin A(M14L, M18L) were found to be significantly sensitive to pepsin and inactivated as a result of degradation. Methionine to Leucine substitution protect the peptide from oxidation but make it more sensitive to gastric conditions since Leucine residues constitute favored cleavage site for pepsin. This is probably one of the explanations for the inconsistency between our results and those reported by Kheadr et al. (2010). Moreover, in current study we used *in vitro* model (static) while Kheadr et al. (2010) performed gastrointestinal digestion in TIM-1 model which is a dynamic model. Therefore, the concentration of enzymes in the two models are different over the digestion period. As a result of extensive posttranslational modification (PTM), class I bacteriocins are more resistant to protease compared to class II bacteriocins (Birri et al., 2012), while in the current study nisin Z was observed to be completely degraded following small intestinal digestion, which is consistent with the previous studies (Heinemann and Williams, 1966; Jarvis and Mahoney, 1969; Gough et al., 2017). Methionine oxidation was observed in nisin Z while it was avoided for pediocin PA-1(M31L) and bactofencin A(M14L, M18L) using Methionine to Leucine substitutions. Furthermore there was a cleavage after the last thioether ring which associated to resistance and has been reported previously (Sun et al., 2009). In a previous study (Naimi et al., 2018), we showed that microcin J25 is highly resistant to the proteolytic enzymes in the stomach, and upon exposure to pancreatin, an enzyme in the small intestine, it was only partially degraded with a minimal loss of activity. The lasso structure of microcin J25 might be the reason for its high stability in extreme conditions, and the partial degradation of microcin J25 in intestinal condition was reported to be due to elastase I, a component of the pancreatin enzyme. Sensitivity of some bacteriocins to GI condition suggests that they could be used as systemic antibiotics (IV treatments) or topical antibiotics for skin or lung. While for oral applications, technologies such as protection and controlled release systems are often necessary to allow them reach their therapeutic target.

Apart from the bioavailability assessment of bacteriocins, their possible interaction with epithelial cells (Caco-2 cells) was evaluated. To evaluate the cytotoxicity effect of bacteriocins in Caco-2 cells, LDH release assay was carried out to assess the membrane integrity of the cells upon exposure to different bacteriocins. The results of this study indicated that membrane integrity remained unaltered in presence of all tested bacteriocins at concentrations up to 400 μg/mL. It should be noted that this concentration is significantly higher than that required to target pathogenic/spoilage bacteria *in vitro*. The MIC values of nisin Z and pediocin PA-1(M31L) against *L. ivanovii* was shown to be 1.65 and 0.09 μg/mL, respectively. While that of microcin J25 was observed to be 0.0356 μg/mL against *S.* Newport, and that of bactofencin A(M14L, M18L) was 5 μg/mL against *S. aureus*. In another study, Shin et al. (2015) showed that nisin Z (Handary, Brussels, Belgium) at concentration up to 200 μg/mL did not exert any toxic effect on human cells relevant to oral cavity which is in line with the current study.

To the best of our knowledge, this is the first study that evaluates the cytotoxicity of several bacteriocins with different structures, mechanisms of actions and spectra of inhibitory activity. In a previous report, pediocin PA-1 and nisin (Sigma-Aldrich sample) were shown to be cytotoxic at high concentrations against Vero and SV40 cells using trypan blue staining viability assay. In fact, SV40 was more sensitive than Vero cells, and at 700 AU/mL (approximately 10–20 mg/mL), pediocin PA-1 and nisin were observed to reduce cell viability to 36 and 50%, respectively; therefore cytotoxicity of pediocin PA-1 was determined to be higher than that of nisin (Murinda et al., 2003). Different commercial nisin origins lead to different IC_50_ values: since these samples are not pure and all contain salts and various contaminating compounds from culture conditions, which may affect concentrations and possibly activity, leading to high discrepancy in the results. In addition commercial nisin samples, contain different nisin analogs (nisin A, nisin C, etc.) which should be taken into consideration. Using MTT assay, Maher and McClean (2006) reported IC_50_ value of commercial nisin A (Nutrition 21) to be 385.7 and 301.5 μg/mL in Caco-2 and HT29 (epithelial cells), respectively (Maher and McClean, 2006). In another study, nisin C (Chrisin^®^) was shown to reduce the viability of Vero cells, and MCF-7 and HepG2 cells to 50% at 45.21 and 352 μg/mL, respectively (Paiva et al., 2012). In a study by Vaucher et al. (2010), EC_50_ value of nisin A (Nisaplin^®^) in Vero cells was determined to be 0.62 μg/mL using LDH cytotoxicity assay. The inconsistency of results between the earlier studies and the current one could be due to impurities and salts in the substances tested. In this study, the purity of nisin and other bacteriocins used was more than 95%, while in the most of the other studies, crude preparation of nisin with high concentrations of salt were used, which can explain the different results. In addition, other factors such as the type of analog used, assay type, cell line, and exposure time can affect the outcome of different investigations.

Hemolytic activity is used for initial toxicity assessment and estimation of therapeutic index. Nisin remains to be the most studied bacteriocin, while there are very few data available for the other bacteriocins. At 33.75 μM (113 μg/mL) concentration, nisin C (Chrisin^®^) was shown to cause 6.6% hemolysis in sheep blood cells (Paiva et al., 2012). Hemolytic activity of nisin A (Nisaplin^®^) at 3.35 μg/mL concentration was reported to be 6% in human red blood cells. In another study by Maher and McClean (2006), hemolytic activity exerted by 230 μM (771 μg/mL) nisin A (Nutrition 21) was shown to be 12.4% in sheep erythrocytes. Moreover, 750 μM nisin (2.5 mg/mL) has shown to cause 10% relative hemolysis against human red blood cells (Begde et al., 2011). In the current study, hemolytic activity caused by nisin Z, pediocin PA-1(M31L), and bactofencin A(M14L, M18L) were shown to be in a dose-dependent manner at concentrations higher than 50 μg/mL, while no lysis was observed in rat erythrocytes exposed to microcin J25 at concentrations up to 400 μg/mL. It is worth to note that differences observed in cytotoxicity effect of nisin Z, pediocin PA-1(M31L), and bactofencin A(M14L, M18L) on RBC (using hemolysis assay) compared to Caco-2 cells (using LDH release assay) might be due to differences in the types of assay, types of medium, incubation time, and different types of cells. Altogether, high antibacterial potency of nisin, pediocin PA-1, bactofencin A(M14L, M18L) and microcin J25 without significant hemolytic effect against red blood cells indicates a high selectivity for bacterial over eukaryotic cells.

Ultimately, this study provides unique scientific data on GI behavior and toxicity of several well-known bacteriocins, which differ in their structural characteristics and mechanisms of action. Using different *in vitro* models, we demonstrated complete degradation of nisin Z, pediocin PA-1(M31L) and bactofencin A(M14L, M18L) in the GI tract, suggesting that these bacteriocins can be safely used in food preservation. Although microcin J25 showed high stability in the GI tract, it did not exert any toxic effect. The data from this study indicate that bacteriocins were non-toxic against eukaryotic cell lines and hemolysis was demonstrated to occur at concentrations significantly higher than their MICs. However, further *in vivo* studies are required to confirm these data and to evaluate the effect of long-term exposure to bacteriocins.

## Data Availability Statement

The original contributions presented in the study are included in the article/Supplementary Material, further inquiries can be directed to the corresponding author.

## Author Contributions

SS, SZ, and IF participated in the experimental design, and responsible for the laboratory analysis, data analysis, and writing. SR participated in writing. FC, YB, and EB participated in experimental design, laboratory analysis, and data analysis. MS participated in experimental design and laboratory analysis. IF obtained the financial support for this study. All authors read and approved the final manuscript. All authors contributed to the article and approved the submitted version.

## Conflict of Interest

The authors declare that the research was conducted in the absence of any commercial or financial relationships that could be construed as a potential conflict of interest.

## Publisher’s Note

All claims expressed in this article are solely those of the authors and do not necessarily represent those of their affiliated organizations, or those of the publisher, the editors and the reviewers. Any product that may be evaluated in this article, or claim that may be made by its manufacturer, is not guaranteed or endorsed by the publisher.

## Footnotes

1
http://bactibase.hammamilab.org/main.php

2
http://gnps.ucsd.edu
