# Supplementary material for: Gastrointestinal Stability and Cytotoxicity of Bacteriocins From Gram-Positive and Gram-Negative Bacteria: A Comparative in vitro Study
Source: Front Microbiol. 2022 Jan 25;12:780355. doi: 10.3389/fmicb.2021.780355 (PMC8824275; doi:10.3389/fmicb.2021.780355)
Supplement: Supplementary file 1 [file Data_Sheet_1.docx]

**Table S1. Degradation products identified for nisin Z.**

The degradation product are labeled according to the nodes assigned in the molecular network are shown on Figure 3. The degradation products N6, N7 and N8 are detected as single nodes on the network. N: no protease, O: oral conditions, G: gastric conditions, I: small intestine conditions, n.d.: not determined.

|  |  | Mw (Da) | RT (min) |  |  |  |
| --- | --- | --- | --- | --- | --- | --- |
| **Nisin Z** | | **3328.5** | **5.6** |  | | |
| Condition | Degradation product |  |  | Mass increment (Da) | Assignment | Segment cleaved off |
| N/O/G | **N1** | 3344.4 | 5.4  5.6 | + 16 | Oxidation (M) | - |
| O/G | **N2** | 3346.5 | 5.2 | + 18 | Hydrolysis in a thioether ring | - |
| N/O/G | **N3** | 3360.5 | 5.3 | + 32 | Oxidation (M) × 2 | - |
| G | **N4** | 2695.1 | 6.2 | - 633 | {Ile1-Ala28} | {Ser29-Lys34} |
| G/I | **N5** | 3032.3 | 5.8 | - 296 | {Ile1-His31} | {Val32-Lys34} |
| G | **N6** | 2895.3 | 6.3 | - 333 | {Ile1-Ile30} | {His31-Lys34} |
| I | **N7** | 1149.6 | 5.9 | - 2179 | {Ile1-Lys12} | {Abu13-Lys34} |
| G | **N8** | 651.4 | 2.9 | - 2577 | {Ser29-Lys34} | {Ile1-Ala28} |
