# Supplementary figures and images for: Gastrointestinal Stability and Cytotoxicity of Bacteriocins From Gram-Positive and Gram-Negative Bacteria: A Comparative in vitro Study
Source: Front Microbiol. 2022 Jan 25;12:780355. doi: 10.3389/fmicb.2021.780355 (PMC8824275; doi:10.3389/fmicb.2021.780355)

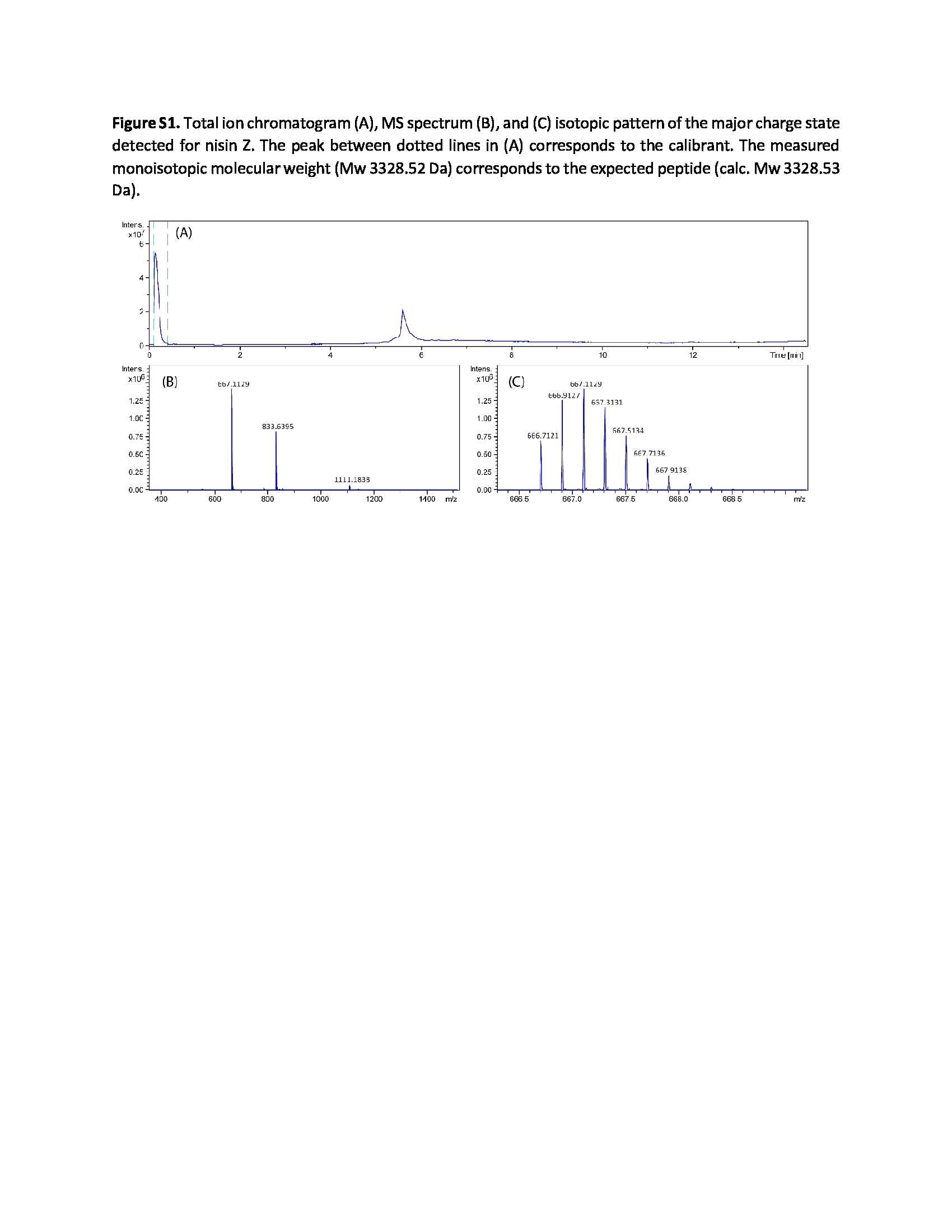

Supplement: Supplementary file 2 [file Image_1.jpeg]

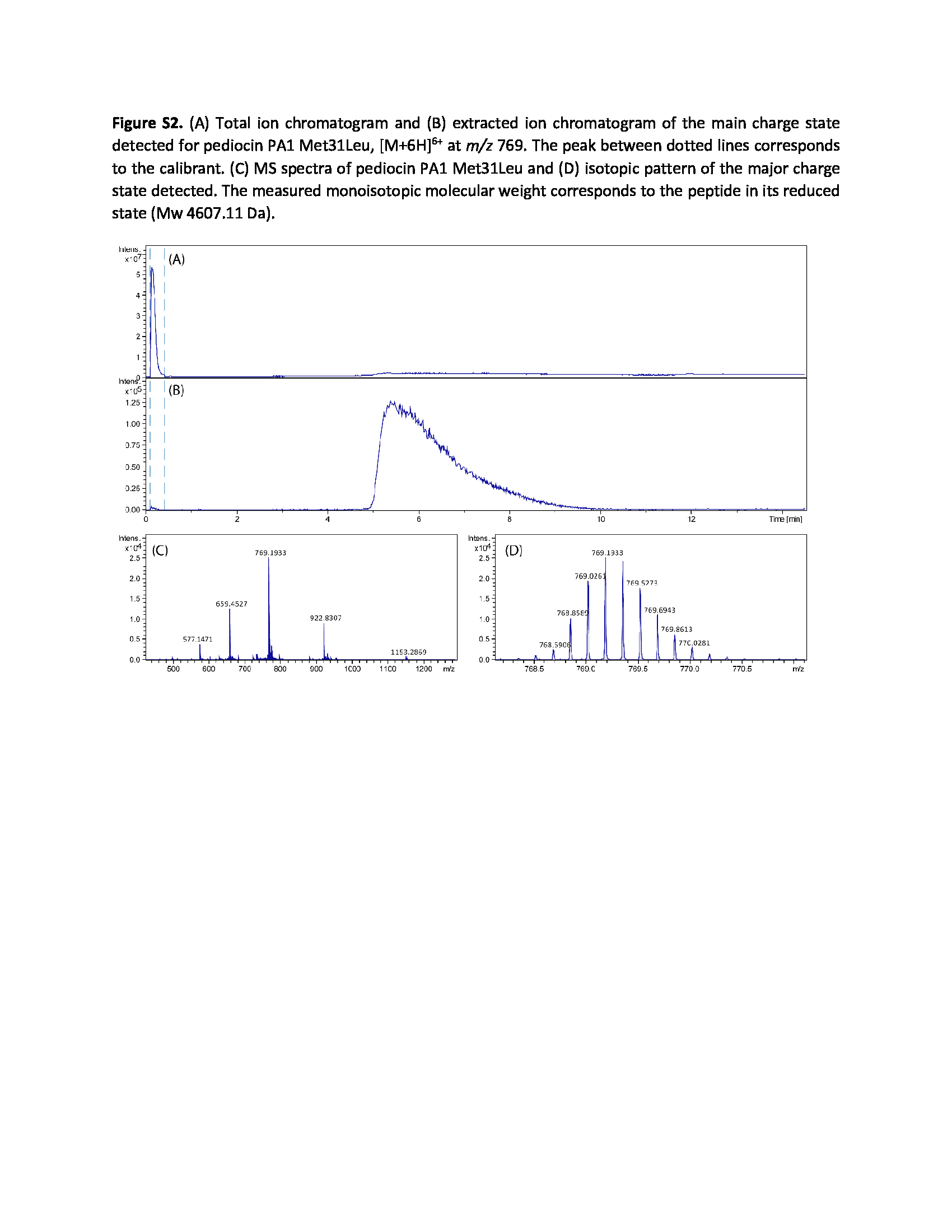

Supplement: Supplementary file 3 [file Image_2.jpeg]

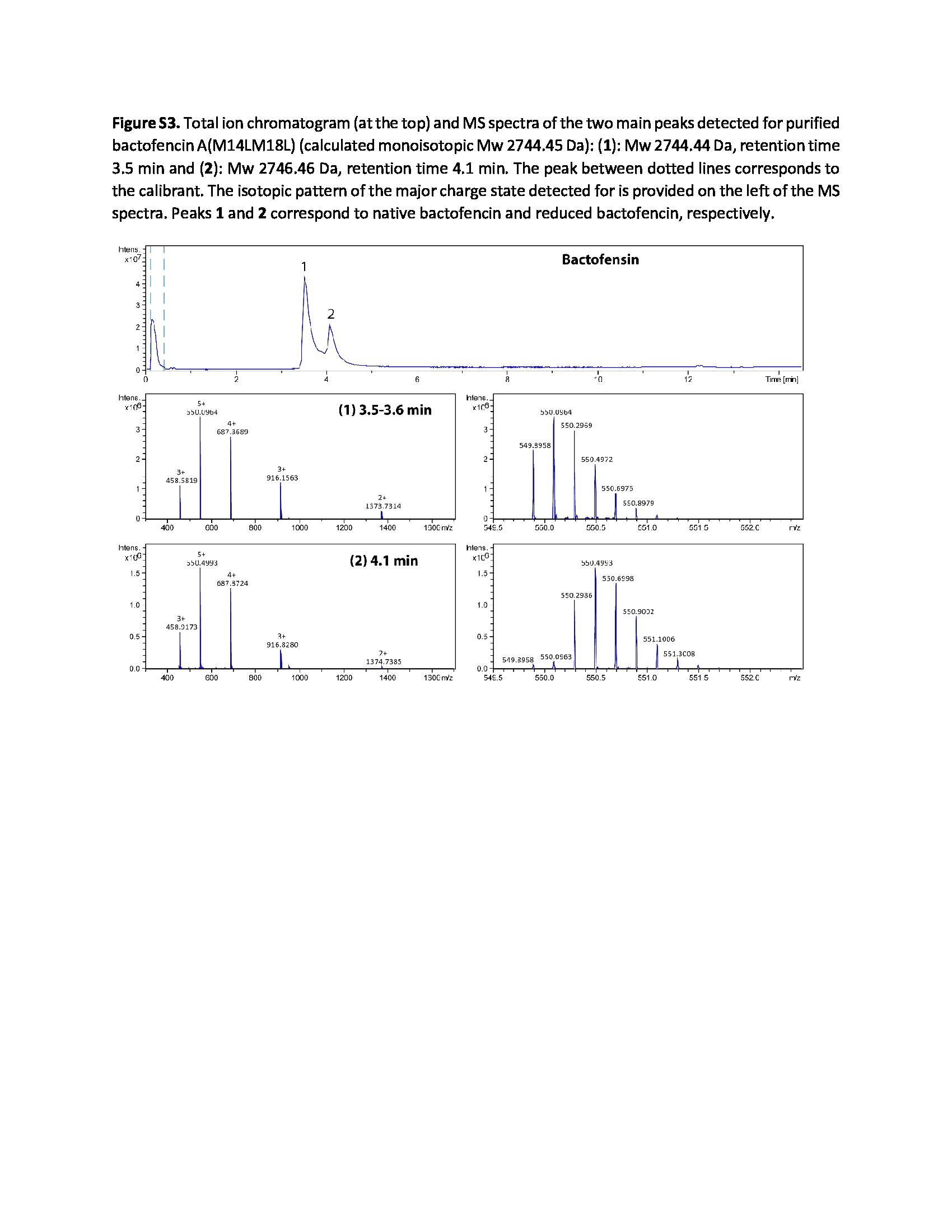

Supplement: Supplementary file 4 [file Image_3.jpeg]

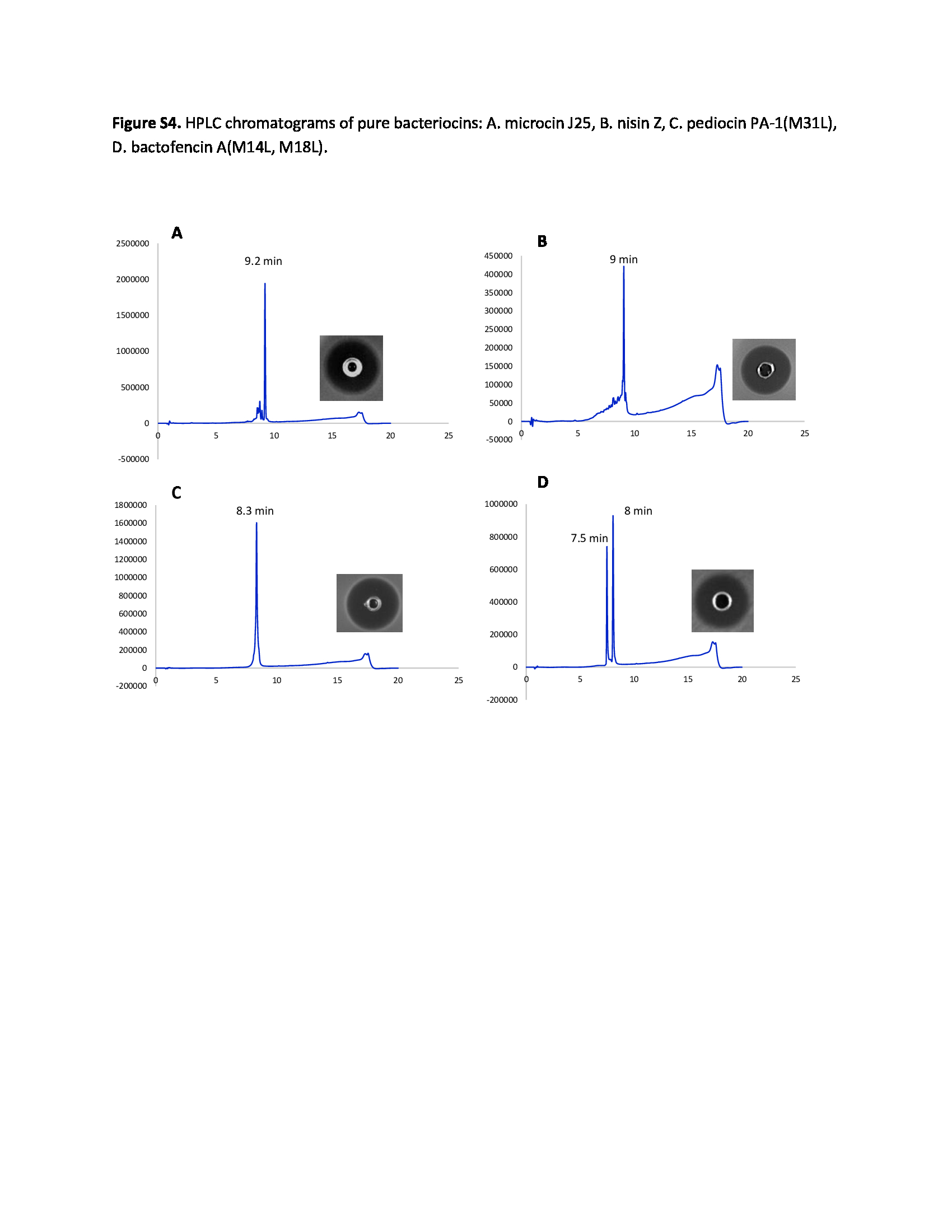

Supplement: Supplementary file 5 [file Image_4.jpeg]

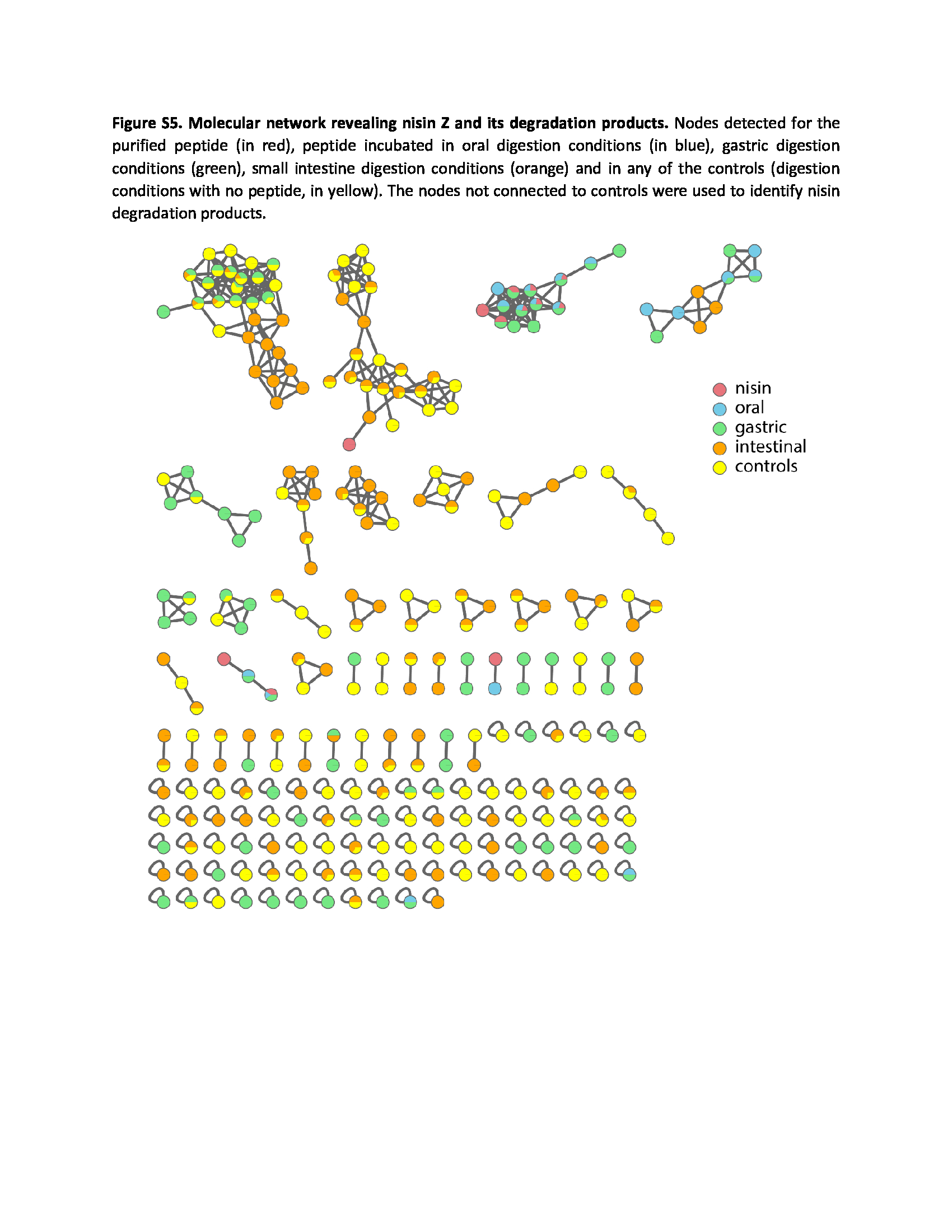

Supplement: Supplementary file 6 [file Image_5.jpeg]

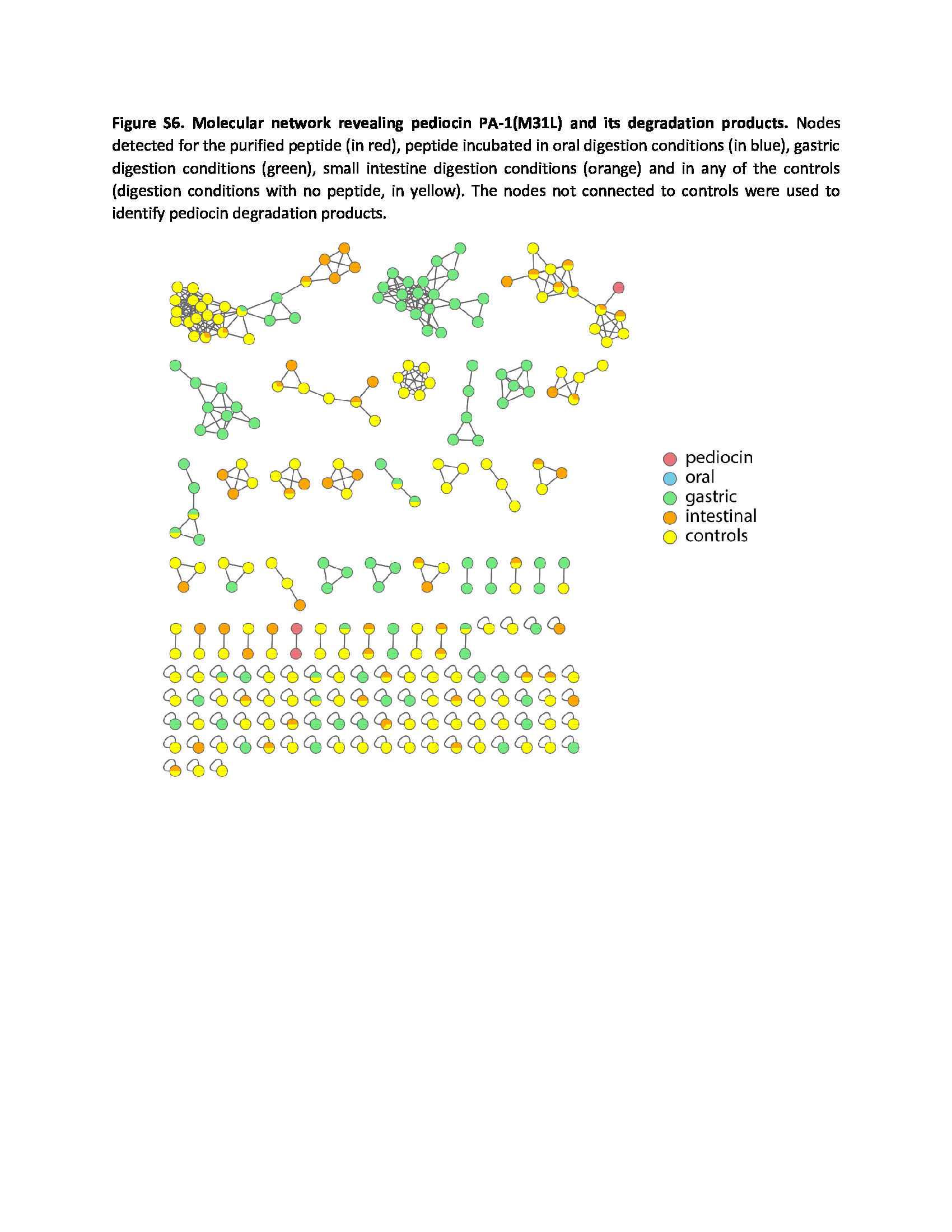

Supplement: Supplementary file 7 [file Image_6.jpeg]

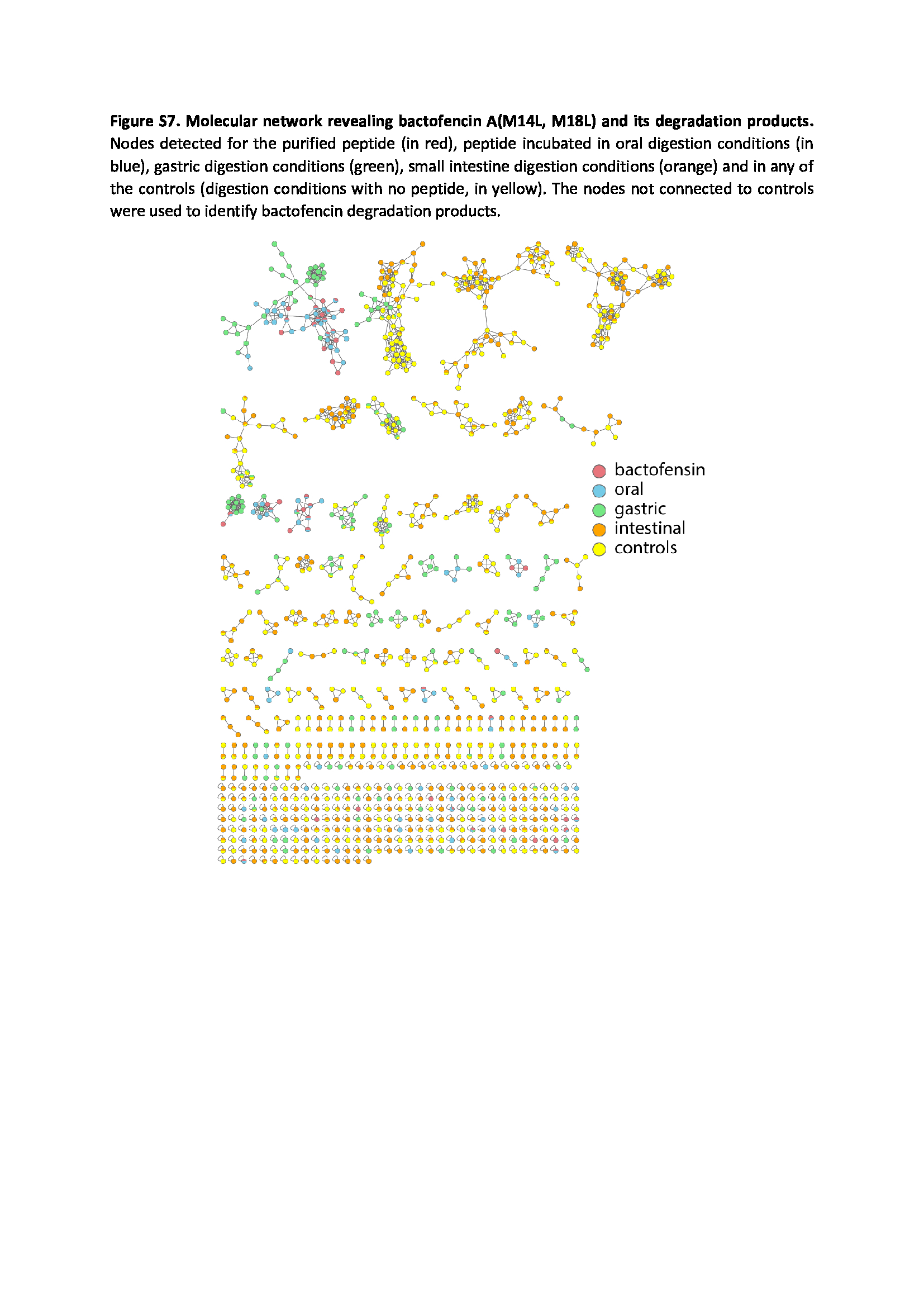

Supplement: Supplementary file 8 [file Image_7.jpeg]

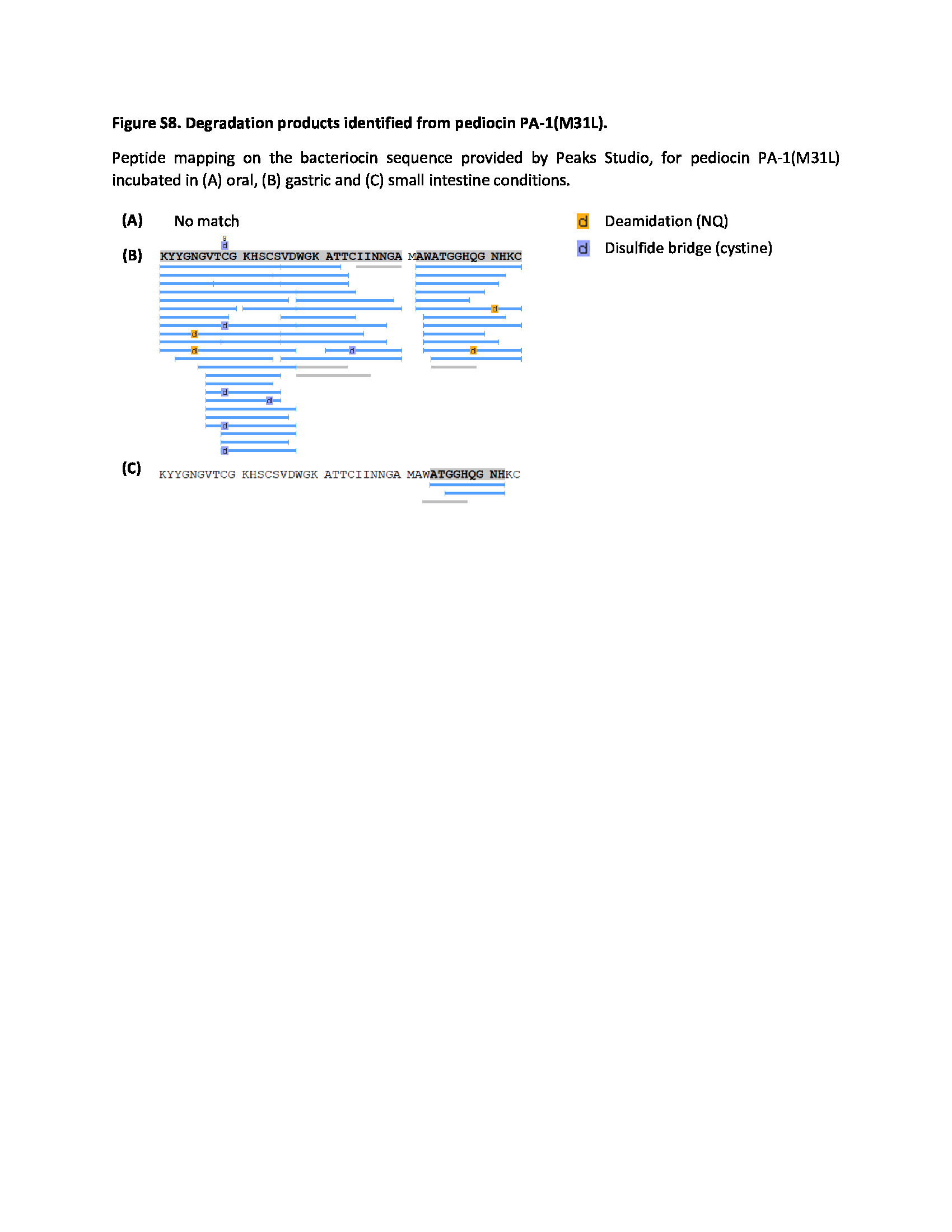

Supplement: Supplementary file 9 [file Image_8.jpeg]

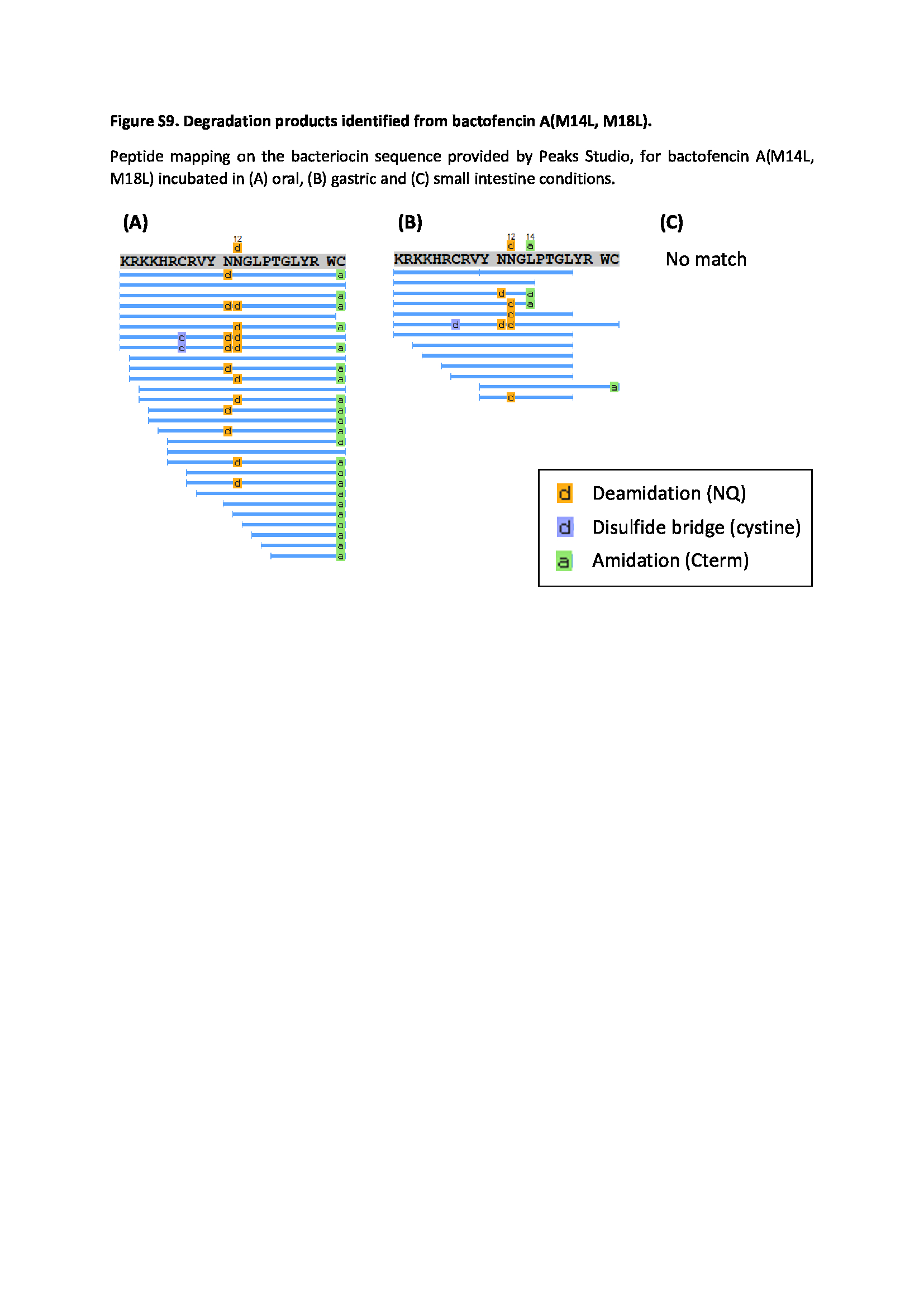

Supplement: Supplementary file 10 [file Image_9.jpeg]
